# Strengthening deep-learning models for intracranial hemorrhage detection: strongly annotated computed tomography images and model ensembles

**DOI:** 10.3389/fneur.2023.1321964

**Published:** 2023-12-29

**Authors:** Dong-Wan Kang, Gi-Hun Park, Wi-Sun Ryu, Dawid Schellingerhout, Museong Kim, Yong Soo Kim, Chan-Young Park, Keon-Joo Lee, Moon-Ku Han, Han-Gil Jeong, Dong-Eog Kim

**Affiliations:** ^1^Department of Public Health, Seoul National University Bundang Hospital, Seongnam, Republic of Korea; ^2^Department of Neurology, Gyeonggi Provincial Medical Center, Icheon Hospital, Icheon, Republic of Korea; ^3^Department of Neurology, Seoul National University Bundang Hospital, Seoul National University College of Medicine, Seongnam, Republic of Korea; ^4^JLK Inc., Artificial Intelligence Research Center, Seoul, Republic of Korea; ^5^Department of Neuroradiology and Imaging Physics, The University of Texas M.D. Anderson Cancer Center, Houston, TX, United States; ^6^Department of Neurosurgery, Seoul National University Bundang Hospital, Seoul National University College of Medicine, Seongnam, Republic of Korea; ^7^Hospital Medicine Center, Seoul National University Bundang Hospital, Seoul National University College of Medicine, Seongnam, Republic of Korea; ^8^Department of Neurology, Nowon Eulji Medical Center, Eulji University School of Medicine, Seoul, Republic of Korea; ^9^Department of Neurology, Chung-Ang University Hospital, Seoul, Republic of Korea; ^10^Department of Neurology, Korea University Guro Hospital, Seoul, Republic of Korea; ^11^Department of Neurology, Dongguk University Ilsan Hospital, Goyang, Republic of Korea; ^12^National Priority Research Center for Stroke, Goyang, Republic of Korea

**Keywords:** deep-learning algorithm, intracranial hemorrhage (ICH), weighted ensemble model, strongly annotated dataset, neuroimaging

## Abstract

**Background and purpose:**

Multiple attempts at intracranial hemorrhage (ICH) detection using deep-learning techniques have been plagued by clinical failures. We aimed to compare the performance of a deep-learning algorithm for ICH detection trained on strongly and weakly annotated datasets, and to assess whether a weighted ensemble model that integrates separate models trained using datasets with different ICH improves performance.

**Methods:**

We used brain CT scans from the Radiological Society of North America (27,861 CT scans, 3,528 ICHs) and AI-Hub (53,045 CT scans, 7,013 ICHs) for training. DenseNet121, InceptionResNetV2, MobileNetV2, and VGG19 were trained on strongly and weakly annotated datasets and compared using independent external test datasets. We then developed a weighted ensemble model combining separate models trained on all ICH, subdural hemorrhage (SDH), subarachnoid hemorrhage (SAH), and small-lesion ICH cases. The final weighted ensemble model was compared to four well-known deep-learning models. After external testing, six neurologists reviewed 91 ICH cases difficult for AI and humans.

**Results:**

InceptionResNetV2, MobileNetV2, and VGG19 models outperformed when trained on strongly annotated datasets. A weighted ensemble model combining models trained on SDH, SAH, and small-lesion ICH had a higher AUC, compared with a model trained on all ICH cases only. This model outperformed four deep-learning models (AUC [95% C.I.]: Ensemble model, 0.953[0.938–0.965]; InceptionResNetV2, 0.852[0.828–0.873]; DenseNet121, 0.875[0.852–0.895]; VGG19, 0.796[0.770–0.821]; MobileNetV2, 0.650[0.620–0.680]; *p* < 0.0001). In addition, the case review showed that a better understanding and management of difficult cases may facilitate clinical use of ICH detection algorithms.

**Conclusion:**

We propose a weighted ensemble model for ICH detection, trained on large-scale, strongly annotated CT scans, as no model can capture all aspects of complex tasks.

## 1 Introduction

Intracranial hemorrhage (ICH) occurs in the intracranial space and encompasses the following six types: epidural hemorrhage (EDH), subdural hemorrhage (SDH), subarachnoid hemorrhage (SAH), intraparenchymal hemorrhage (IPH), intraventricular hemorrhage (IVH), and mixed hemorrhage. A timely and accurate diagnosis of ICH and its subtypes is critical for treatment, because of the high mortality and morbidity. In addition, assessing the location and extent of ICH is important for outcome prediction. However, neuroradiology training requires a significant investment of time and resources; accordingly, neuroradiologists are scarce in many countries (1). Without neuroradiologists’ assistance, doctors who see ICH patients often misdiagnose (2).

Deep-learning algorithms have recently made progress in accurately detecting ICH on CT scans (3). Several studies have investigated deep-learning algorithms in detecting ICH. Lee et al. (4) trained a simple artificial neural network model on 250 brain CT scans, including 150 cases of ICH. The model had a suboptimal sensitivity of 0.83 and specificity of 0.76. This study included a relatively small number of cases and did not perfectly detect small lesions. Kuo et al. (3) trained ResNet on 4,396 head CT scans, including 1,131 cases of ICH. Two radiologists verified the pixel-wise labels for acute ICH. The model exhibited an AUC of 0.98, but they did not externally validate the model. Sage et al. (5) proposed a model that used two branch ResNet-50 architectures to train 3-channel images with different Hounsfield unit windows and 3-channel images with consecutive slices, and determined the final result by random forest. The model showed an accuracy of 0.891 for SDH, 0.743 for EDH, 0.933 for IPH, 0.967 for IVH, and 0.897 for SAH. Salehinejad et al. (6) trained the SE-PreNetXt architecture to demonstrate ICH detection on real-world data. They performed external validation on an emergency department dataset without exclusion and achieved the excellent AUC values except for EDH cases. The relatively low accuracy for EDH in these two studies might have been due to the lack of EDH cases in the Radiological Society of North America (RSNA) training dataset (7). Taken together, although a number of novel methods have been proposed, AI performance may be hampered by relatively low accuracy for small lesions or low accuracy for the particular subtype due to its paucity in the training datasets. Insufficient data or weak annotations have been employed in the majority of published research (3, 7, 8). Establishing large datasets with expert annotations is difficult because it requires a lot of effort and resources (9). In the classification-based deep learning using images with labels that are relatively easy to obtain (presence vs. absence of ICH), saliency maps could not locate the exact location of lesions (10). Whether ICH detection performance improves when training on a strongly annotated dataset with pixel-level annotations needs to be investigated.

Another unresolved problem with ICH detection algorithms to date is the difficulty of differentiating between distinct subtypes, especially SDH and SAH (11). ICH subtypes are defined based on the location of hemorrhages within the anatomic structures, such as the brain parenchyma, ventricle, dura, and subarachnoid space. Therefore, each subtype has a distinct morphology, location, and spatial correlation with adjacent tissue. To be specific, SDH is more likely to present in the subacute stage and has reduced CT attenuation, similar to that of brain tissue. SAH may also appear isoattenuation if there is only a small amount of blood mixed with cerebrospinal fluid (12). Moreover, a pseudo-SAH is not an uncommon finding; it manifests as high-attenuation areas along the basal cisterns, Sylvian fissure, tentorium cerebelli, or cortical sulci. Seyam et al. (11) demonstrated that a commercially available deep learning algorithm used in the real world exhibited particularly low accuracy for SDH and SAH, highlighting the need to acknowledge the limitations of AI tools. Wang et al. proposed an AI algorithm consisting of a 2D CNN classifier and two sequence models and trained on 25,000 CT scans from the RSNA dataset. The proposed model accurately classified ICH subtypes and showed AUCs of 0.964 and 0.949 for two external validation datasets. However, the model had relatively low predictions for small SDH (13). Deep learning algorithms with better performance for all ICH subtypes are needed, and we propose a weighted ensemble model in this study.

In this study, we investigated the following two hypotheses. (1) We investigated whether a deep-learning model performs better if it is trained on a large annotated dataset with slice-wise manual segmentation, compared to the one trained on a weakly annotated dataset. (2) We aimed to determine if a weighted ensemble model, which integrates multiple models trained on distinct datasets of ICH subtypes, could effectively minimize prediction errors and preserve the robustness of each individual model across ICH subtypes and sizes. In addition, six experts reviewed challenging ICH cases after external testing of the final model.

## 2 Methods

### 2.1 Model development with weakly and strongly annotated datasets

#### 2.1.1 Datasets

##### 2.1.1.1 Weakly annotated dataset

We used open data from the RSNA comprising 27,861 brain CT scans (3,528 hemorrhages). Per slice, the neuroradiologists labeled the presence/absence of a hemorrhage without spatial annotation (7).

##### 2.1.1.2 Strongly annotated dataset

We used 53,045 brain CT scans (7,013 with and 46,032 without ICH) from the AI-Hub directed by the Korean National Information Society Agency.^1^ The AI-Hub dataset was collected from six Korean university hospitals in 2020 as part of a large-scale data collection initiative for cerebrovascular disease. Each hospital’s neuroradiologist interpreted the CT scans, labeled the presence of hemorrhage per slice, and manually segmented the outline of the hemorrhage.

##### 2.1.2 Training and validation dataset

**Figure 1:**
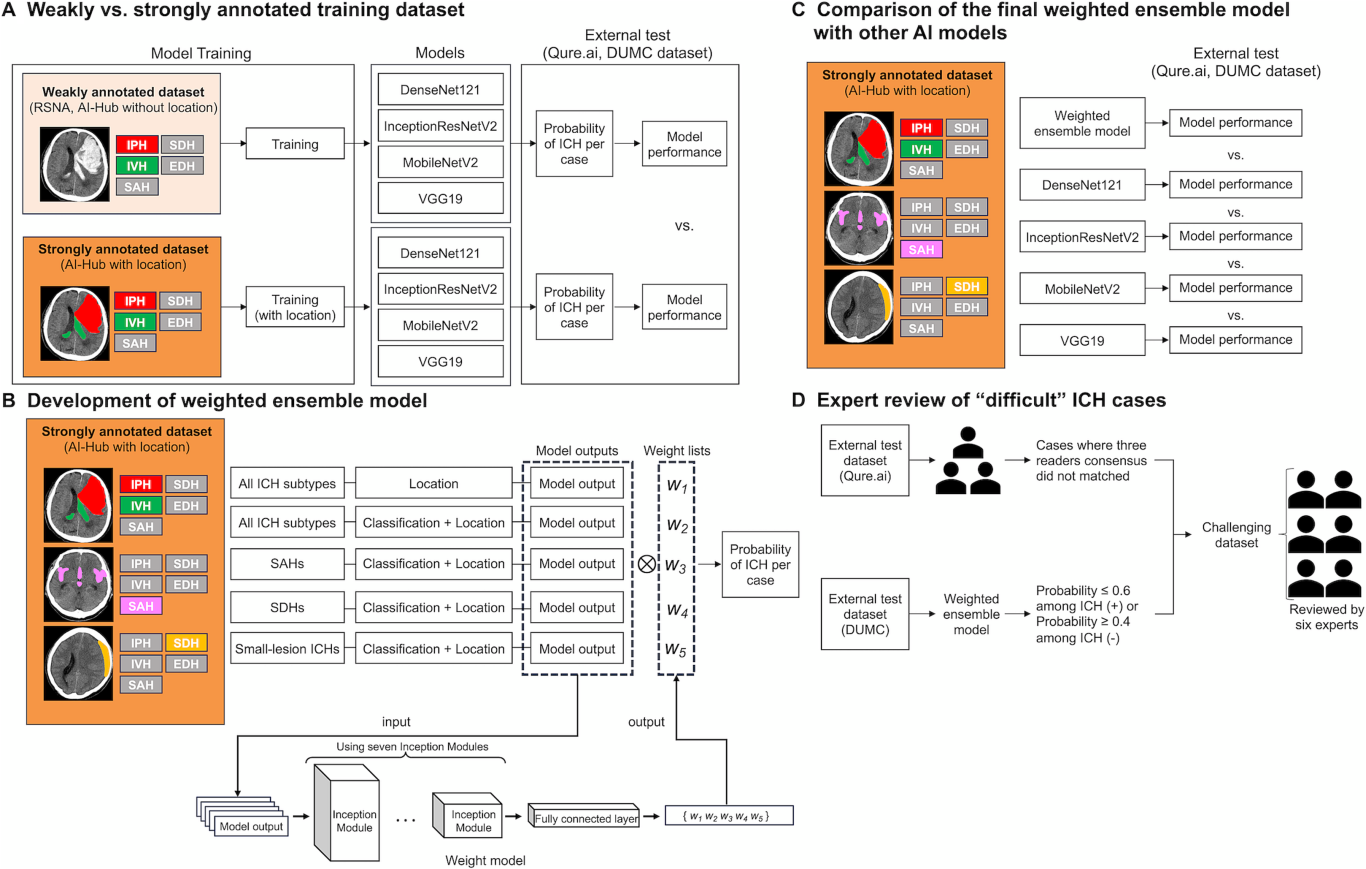
A schematic of this study. **(A)** Comparison of the performances of models trained on weakly annotated datasets with models trained on a strongly annotated dataset. **(B)** Development of the weighted ensemble model. **(C)** Comparison of the final weighted ensemble model with other AI models. **(D)** Expert review of “difficult” ICH cases. ICH, intracranial hemorrhage; RSNA, the Radiological Society of North America; IPH, intraparenchymal hemorrhage; SDH, subdural hemorrhage; IVH, intraventricular hemorrhage; EDH, epidural hemorrhage; SAH, subarachnoid hemorrhage; DUMC, Dongguk University Medical Center.

A schematic overview and summary of each deep-learning model used in this study are shown in Figure 1 and Supplementary Table S1, respectively. To compare the performance of the deep-learning models trained on weakly and strongly annotated datasets, and to account for different data sizes, we randomly selected the same number of slices with and without hemorrhage (*n* = 6,500 each) from the RSNA and AI-Hub datasets. For CT scans with hemorrhage, the same number of slices as those of IPH, IVH, SDH, EDH, and SAH (*n* = 1,300) were included, i.e., the hemorrhagic types were balanced. All training dataset images were pre-processed into four-channel input data (Supplementary Figure S1).

##### 2.1.3 External test dataset

Two datasets were used for the external testing of the deep-learning models. The first dataset comprised 600 brain CT scans (327 with and 273 without ICH) from a tertiary hospital in Korea [Dongguk University Medical Center (DUMC)]. The second was an open dataset (Qure.ai) comprising 386 CTs (160 with and 226 without ICH). A vascular neurologist and neuroradiologist performed consensus labeling for the DUMC dataset, and three neuroradiologists labeled the Qure.ai dataset using a majority vote. The study protocol was designed in accordance with the Declaration of Helsinki. This study was approved by the institutional review board of DUMC and JLK Inc. (No. DUIH 2018–03-018 and 20,220,407–01), and the requirement to obtain informed consent was waived due to data anonymization, inability to contact patients, and minimal risk.

##### 2.1.4 Pre-processing

A four-channel input image was used to develop the model. As previously described (5, 14–16), CT windowing was used to generate three different images:(1) a stroke window (width 40 and level 40), (2) brain window (width 80 and level 40), and (3) bone window (width 3,000 and level 500). A 3D U-net model developed in-house was used to strip the skull (17). We applied the skull stripping model to the 2) brain window image to create a 4) brain window with skull stripping (Supplementary Figure S1).

##### 2.1.5 Comparison of models trained with weakly and strongly annotated datasets

DenseNet121 (18), InceptionResNetV2 (19), MobileNetV2 (20), and VGG19 (21) were used for model development to compare deep-learning models trained on datasets with weak and strong annotations. For models using weakly annotated datasets, we used slice-wise hemorrhage labeling for both the RSNA and AI-Hub datasets (Supplementary Figures S2A,B). For classification loss, we compared the slice-wise model output and ground-truth labeling. The same input image was fed into the deep-learning model trained on the strongly annotated AI-Hub dataset (Supplementary Figure S2C). Our aim was to use strong annotation to improve classification performance. Thus, we utilized them to train the saliency map to locate the exact lesions more precisely. We extracted the saliency map from the last convolutional layer of each deep-learning model, compared it to the ground-truth hemorrhage segmentation, and computed the segmentation loss in addition to the classification loss to train the hemorrhage location.

We tested each model on an external test dataset and calculated sensitivity, specificity, and AUC and the threshold of 0.5 as the model’s performance. We used 500 bootstrap replications to calculate 95% confidence intervals. We used the DeLong test for AUC comparison (22).

### 2.2 Ensemble model

#### 2.2.1 Training and test dataset

**Figure 2:**
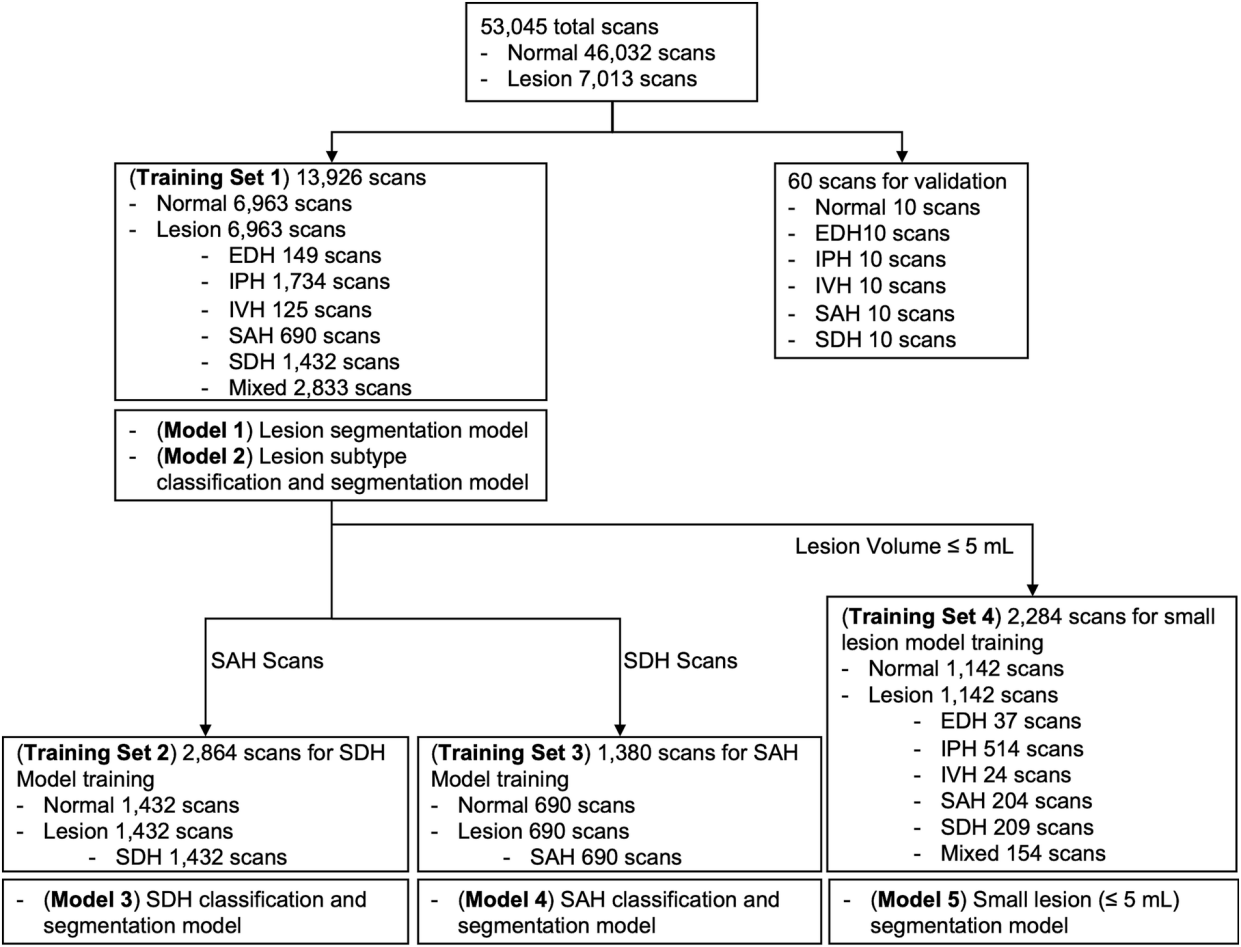
Summary of each deep-learning model and the training dataset used. EDH, epidural hemorrhage; IPH, intraparenchymal hemorrhage; IVH, intraventricular hemorrhage; SAH, subarachnoid hemorrhage; SDH, subdural hemorrhage.

Considering class imbalance, we randomly selected an equal number of brain CT scans with and without ICH from the AI-Hub dataset (6,963 each). For each training dataset consisting of only SDH, SAH, and small-lesion cases, we also balanced the number of normal and lesion cases equally (SDH, 1,432 each; SAH, 690 each; small lesion, 1,142 each). We trained five U-net based segmentation models (Supplementary Figure S3): Lesion segmentation model using all training datasets (Model 1), lesion subtype pre-trained segmentation model using all training datasets (Model 2), SDH model (Model 3), SAH model (Model 4), and small lesion (≤ 5 mL) model (Model 5). A summary of each model and the training dataset is shown in Figure 2. The DUMC and Qure.ai datasets were used for external testing.

#### 2.2.2 Ensemble base models

Five deep-learning models were trained using 2D U-net with the Inception module (Supplementary Figure S3) (23, 24). For the lesion subtype pre-trained segmentation model (Model 2), a pre-trained model in which down-sampling layers of U-net were pre-trained using hemorrhage subtype labeling was used. The Dice loss function, Adam optimizer, and a learning rate of 1e-4 were used for model training. The hyperparameters of each ensemble base model used in this study are shown in Supplementary Table S2.

To determine whether the ensemble of base models (Models 1–5) improved the performance of hemorrhage detection in SAH, SDH, and small hemorrhage cases, we combined the base models and evaluated the performance of each combination model. From the two external test datasets, we extracted SAH, SDH, and small hemorrhage cases (with the same number of normal CT scans) to test the combination model.

#### 2.2.3 Weighted ensemble model

**Figure 3:**
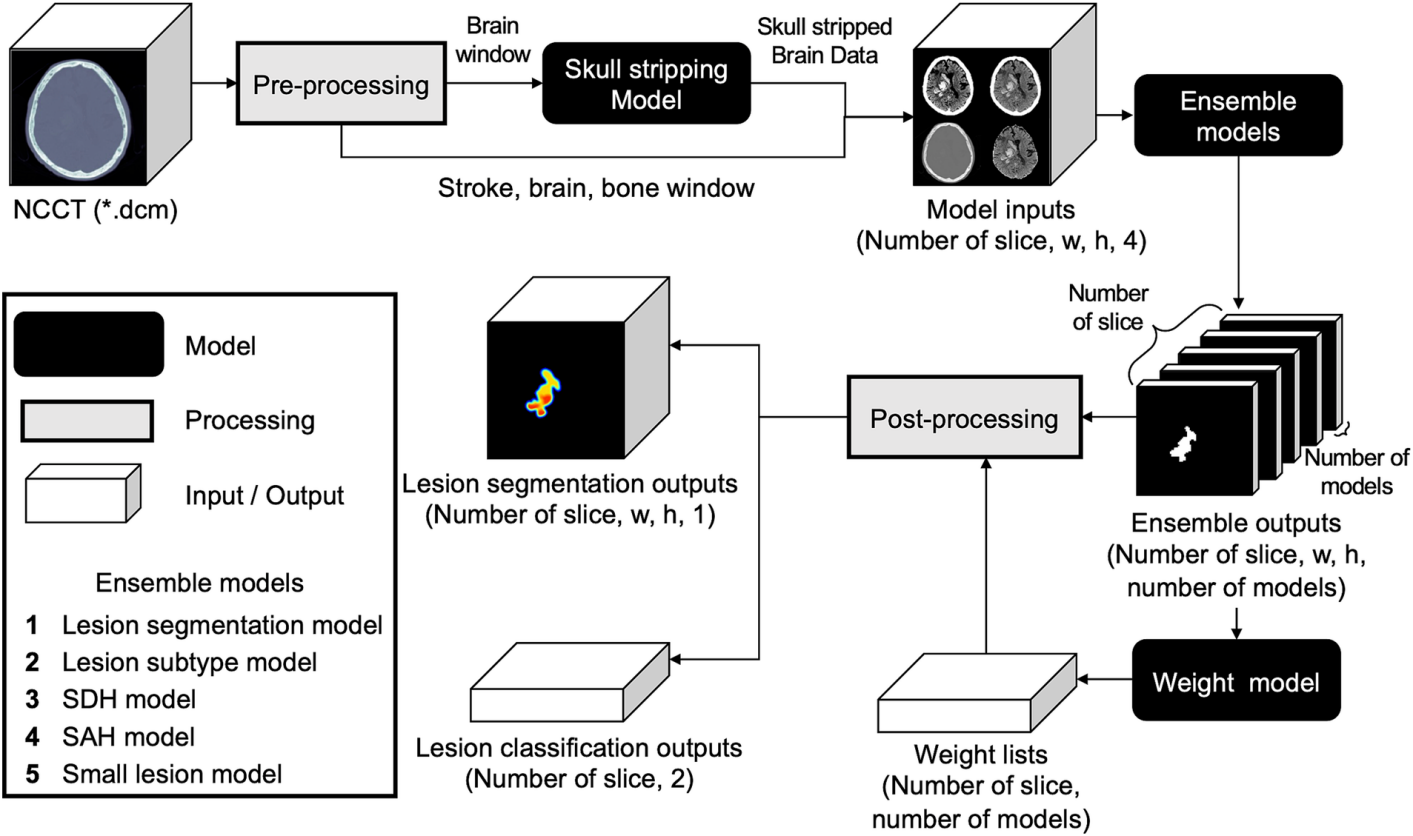
Comprehensive workflow of the developed weighted ensemble model. The process includes pre-processing, skull stripping, ensembling models with weight model, and post-processing.

To ensemble the five base models using distinct datasets, their outputs needed to be assigned appropriate weight values according to the input data. Hence, we developed an additional weight model using input data comprising five-channel segmentation results from five base models, ranging from zero to one. Using random initiative weight values, the model was trained to select the weight values that minimized the Dice loss between the predicted segmentation at the pixel probability threshold of 0.5 and ground-truth segmentation (Figure 3; Supplementary Figure S4).

#### 2.2.4 Post-processing

Five different hemorrhage detection models and the weighted ensemble model yielded five segmentation outputs and weight values per slice. The segmentation outputs and weight values were then multiplied using the following equation: The highest pixel probability value was selected as the maximal probability at the slice level (max[HemorrhageSlice]).


$$f\left(\text{Slice}\right)=\max\left(\frac{\sum_{i=1}^{5}\text{SegOu}t_{i}\left(\text{Pixel}\right)*\text{WeightOu}t_{i}\left(\text{Pixel}\right)}{5}\right)$$

SegOut_i_ = output of each segmentation model; WeightOut_i_ = weight values for each segmentation model.

We used the following equation to calculate the hemorrhage probability per case, in which max (HemorrhageSlice) indicates the maximal slice probability.


$$\begin{array}{c} \text{Probablity}\\ \text{of}\;\text{Case} \end{array}=\frac{\sum_{i=1}^{\text{number}\;\text{of}\;\text{slice}\;\text{with}\;\text{Hemorrhage}}\text{HemorrhageSlic}e_{i}}{\text{number}\;\text{of}\;\text{slice}\;\text{with}\;\text{Hemorrhage}}$$

### 2.3 Review of “difficult” ICH cases

After external testing of the weighted ensemble model, we defined the difficult ICH cases for expert reviews. “Difficult-for-AI” cases were chosen from the DUMC dataset when a) the probability of lesion ≤0.6 among cases annotated as hemorrhage or b) the probability of lesion ≥0.4 among cases annotated as no hemorrhage. “Difficult-for-humans” cases were selected from the Qure.ai dataset if the three annotators had not unanimously agreed with the ground truth during the initial labeling process.

Six neurology experts with six to eighteen years of clinical experience re-annotated the presence vs. absence of ICH in these two types of difficult images. The sensitivity, specificity, and accuracy of the weighted ensemble model and each expert were calculated. The inter-rater agreement among the experts was also calculated. If the ground truth and the opinions of six experts did not concur, a consensus meeting was held to amend the ground truth with a majority (4 or higher) vote. After the consensus meeting, the sensitivity, specificity, and accuracy of the weighted ensemble model and those of the six experts were re-calculated. Cases where the weighted ensemble model made incorrect predictions were subjected to qualitative assessments.

## 3 Results

### 3.1 Baseline characteristics of the datasets

The AI-Hub dataset, in which the presence of each hemorrhage subtype was labeled per slice and the hemorrhage was manually segmented, had 53,045 cases (mean 57.5 years, 47.5% female), and 13.2% (7,013) had ICH. In the RSNA dataset with the presence of each ICH subtype being labeled per slice, 39.6% (7,449) of 18,938 cases had ICH. Table 1 shows the proportion of each ICH subtype in the AI-Hub and RSNA datasets. The baseline characteristics of the external datasets from DUMC and Qure.ai are also presented in Table 1.

**Table 1:** Baseline characteristics of the datasets.

	Training dataset		Test dataset	
	AI-Hub	RSNA	DUMC	Qure.ai
Country	South Korea	Unites States	South Korea	India
Number of cases	53,045	18,938	600	386
Age (mean ± S.D.)	57.5 ± 19.9	N/A	65.7 ± 14.9	N/A
Female Sex	25,185 (47.5%)	N/A	213 (35.5%)	N/A
Normal	46,032 (86.8%)	11,439 (60.4%)	273 (45.5%)	226 (58.5%)
ICH	7,013 (13.2%)	7,499 (39.6%)	327 (54.5%)	160 (41.5%)
IPH	1,744 (24.9%)^†^	1,008 (13.4%)^†^	221 (67.6%)^†^	45 (28.1%)^†^
IVH	135 (1.9%)^†^	239 (3.2%)^†^	8 (2.4%)^†^	0 (0%)^†^
EDH	159 (2.3%)^†^	73 (1.0%)^†^	1 (0.3%)^†^	0 (0%)^†^
SDH	1,442 (20.6%)^†^	1,298 (17.3%)^†^	15 (4.6%)^†^	0 (0%)^†^
SAH	700 (10.0%)^†^	456 (6.1%)^†^	82 (25.1%)^†^	0 (0%)^†^
Mixed	2,833 (40.4%)^†^	4,425 (59.0%)^†^	0 (0%)^†^	115 (71.9%)^†^

^†^The percentages indicate the proportion of each subtype to the total number of lesions. RSNA, the Radiological Society of North America; DUMC, Dongguk University Medical Center; ICH, intracranial hemorrhage; IPH, intraparenchymal hemorrhage; IVH, intraventricular hemorrhage; EDH, epidural hemorrhage; SDH, subdural hemorrhage; SAH, subarachnoid hemorrhage.

### 3.2 Comparison of models trained with weakly vs. strongly annotated datasets

A dataset with strong annotations (AI-Hub dataset with location information) and two datasets with weak annotations (AI-Hub dataset without location information and RSNA dataset) were utilized for the training of four well-known deep-learning networks: DenseNet121, InceptionResNetV2, MobileNetV2, and VGG19. We tested four trained models on a composite of the DUMC and Qure.ai datasets. When trained using the RSNA dataset, the accuracies of DenseNet121, InceptionResNetV2, MobileNetV2, and VGG19 was 0.771 (95% C.I. 0.767–0.775), 0.770 (95% C.I. 0.766–0.774), 0.649 (95% C.I. 0.645–0.653), and 0.708 (95% C.I. 0.704–0.712), respectively. When trained using the AI-Hub dataset without location information, the accuracies were 0.812 (95% C.I. 0.809–0.816), 0.810 (95% C.I. 0.807–0.814), 0.645 (95% C.I. 0.641–0.650), and 0.707 (95% C.I. 0.705–0.711), respectively. When trained using the AI-Hub dataset with location information, the accuracies of all deep-learning networks except for DenseNet121 improved significantly, with the values being 0.756 (95% C.I. 0.753–0.761), 0.818 (95% C.I. 0.812–0.820), 0.658 (95% C.I. 0.655–0.664), and 0.862 (95% C.I. 0.859–0.865), respectively (Table 2; Supplementary Figure S5).

**Table 2:** Comparison of models trained with weakly and strongly annotated datasets.

Value (95% C.I.)	RSNA dataset			AI-Hub without location dataset			AI-Hub with location dataset		
	AUC	Sensitivity	Specificity	AUC	Sensitivity	Specificity	AUC	Sensitivity	Specificity
**DenseNet121**	0.771 (0.767–0.775)	0.616 (0.611–0.620)	0.754 (0.745–0.765)	0.812 (0.809–0.816)	0.554 (0.548–0.558)	0.887 (0.880–0.895)	0.756 (0.753–0.761)	0.693 (0.688–0.698)	0.657 (0.647–0.668)
P for AUC difference	Reference			*p* = 0.0003 (*0.0039)			*p* < 0.0001 (*0.0030)		
**InceptionResNetV2**	0.770 (0.766–0.774)	0.623 (0.618–0.628)	0.724 (0.716–0.736)	0.810 (0.807–0.814)	0.658 (0.654–0.663)	0.825 (0.817–0.834)	0.818 (0.812–0.820)	0.745 (0.741–0.750)	0.725 (0.715–0.735)
P for AUC difference	Reference			*p* < 0.0001 (*0.0031)			*p* < 0.0001 (*0.0031)		
**MobileNetV2**	0.649 (0.645–0.653)	0.599 (0.595–0.605)	0.600 (0.589–0.611)	0.645 (0.641–0.65)	0.599 (0.595–0.605)	0.608 (0.598–0.620)	0.658 (0.655–0.664)	0.708 (0.704–0.713)	0.499 (0.488–0.510)
P for AUC difference	Reference			*p* = 0.4221 (*0.0047)			*p* = 0.0227 (*0.0040)		
**VGG19**	0.708 (0.704–0.712)	0.569 (0.564–0.574)	0.754 (0.745–0.764)	0.707 (0.705–0.711)	0.460 (0.455–0.465)	0.876 (0.869–0.884)	0.862 (0.859–0.865)	0.816 (0.812–0.820)	0.731 (0.721–0.741)
P for AUC difference	Reference			*p* = 0.6526 (*0.0032)			*p* < 0.0001 (*0.0029)		

Area under the curve (AUC), sensitivity, and specificity of four deep-learning networks trained on RSNA dataset, AI-Hub dataset without location information, and AI-Hub dataset with location information were shown. The AUC of each of the four deep-learning networks trained on the RSNA dataset was set as a reference, and the AUCs of the remaining models were compared using the DeLong test. C.I., confidence interval. *Standard error.

### 3.3 Development of a weighted ensemble model

To improve the detection of SDH, SAH, and tiny lesions, which is regarded to be challenging, we designed a weighted ensemble model using multiple distinct datasets that not only had strong annotations but also reflected the various ICH features.

We first generated “Ensemble basic,” a weighted ensemble of two models, a lesion segmentation model (Model 1) and a subtype classification/segmentation model (Model 2), which were trained using CT images encompassing all ICH subtypes. Overall ICH detection accuracy measured by using a composite dataset of DUMC and Qure.ai, was 0.938 (95% C.I. 0.922–0.953, Table 3). The accuracy for SDH and SAH cases was, respectively, 0.893 (95% C.I. 0.865–0.919) and 0.944 (95% C.I. 0.942–0.945). Next, to further increase the accuracy in the diagnosis of SDH, SAH, and small lesions, we additionally developed models 3, 4, and 5, which were, respectively, trained on only SDH cases, only SAH cases, and only small lesions ≤5 mL. We then investigated whether the model performance was improved by combining Models 3, 4, or 5 with the weighted ensemble models for Models 1 and 2. “Ensemble SDH” showed an increased accuracy for SDH compared to “Ensemble basic,” from 0.893 (95% C.I. 0.865–0.919) to 0.927 (95% C.I. 0.903–0.948; *P* for AUC difference = 0.0002). For SAH, “Ensemble SAH” showed a comparable accuracy to “Ensemble basic” (0.952 [95% C.I. 0.951–0.954] vs. 0.944 [95% C.I. 0.942–0.945], *P* for AUC difference = 0.2439). “Ensemble small lesions” showed a comparable accuracy for total ICH compared to “Ensemble basic” (0.946 [95% C.I. 0.931–0.960] vs. 0.938 [95% C.I. 0.922–0.953], *P* for AUC difference = 0.1180). Finally, we developed a final weighted ensemble model that ensembles all models 1 to 5 and showed a significantly higher accuracy for total ICHs (0.951 [95% C.I. 0.937–0.964], *P* for AUC difference = 0.0379), compared to “Ensemble basic”.

**Table 3:** The structures of five weighted ensemble models and accuracies for all cases, SDH cases, and SAH cases.

	Combination of the models	All (492 ICH, 494 normal)			SDH (43 SDH, 494 normal)			SAH (117 SAH, 494 normal)		
		AUC	Sensitivity	Specificity	AUC	Sensitivity	Specificity	AUC	Sensitivity	Specificity
**Ensemble basic**	1 + 2	0.938 (0.922–0.953)	0.982 (0.966–0.992)	0.702 (0.662–0.744)	0.893 (0.865–0.919)	0.977 (0.877–0.999)	0.702 (0.662–0.744)	0.944 (0.942–0.945)	0.992 (0.953–1.000)	0.702 (0.662–0.744)
P for AUC difference		Reference			Reference			Reference		
**Ensemble SDH**	1 + 2 + 3	0.936 (0.920–0.951)	0.957 (0.935–0.973)	0.781 (0.757–0.830)	0.927 (0.903–0.948)	0.977 (0.877–0.999)	0.781 (0.757–0.830)	0.941 (0.940–0.943)	0.992 (0.953–1.000)	0.781 (0.757–0.830)
P for AUC difference		*p* = 0.7612 (*0.0060)			*p* = 0.0002 (*0.0092)			*p* = 0.6537 (*0.0071)		
**Ensemble SAH**	1 + 2 + 4	0.944 (0.928–0.958)	0.963 (0.943–0.978)	0.777 (0.753–0.826)	0.868 (0.837–0.896)	0.860 (0.721–0.947)	0.777 (0.753–0.826)	0.952 (0.951–0.954)	0.983 (0.940–0.998)	0.777 (0.753–0.826)
P for AUC difference		*p* = 0.3337 (*0.0057)			*p* = 0.1695 (*0.0186)			*p* = 0.2439 (*0.0069)		
**Ensemble small lesions**	1 + 2 + 5	0.946 (0.931–0.960)	0.970 (0.950–0.983)	0.775 (0.738–0.813)	0.874 (0.843–0.901)	0.860 (0.721–0.947)	0.775 (0.738–0.813)	0.950 (0.948–0.951)	0.983 (0.940–0.998)	0.775 (0.738–0.813)
P for AUC difference		*p* = 0.1180 (*0.0053)			*p* = 0.2023 (*0.0155)			*p* = 0.3621 (*0.0063)		
**Ensemble all**	1 + 2 + 3 + 4 + 5	0.951 (0.937–0.964)	0.943 (0.919–0.962)	0.826 (0.794–0.862)	0.893 (0.864–0.918)	0.884 (0.749–0.961)	0.826 (0.794–0.862)	0.958 (0.958–0.960)	0.974 (0.927–0.995)	0.826 (0.794–0.862)
P for AUC difference		*p* = 0.0379 (*0.0064)			*p* = 0.9845 (*0.0194)			*p* = 0.0644 (*0.0077)		

The area under the curves (AUCs) of “Ensemble basic” for total ICH, SDH, and SAH were set as references, and the AUCs of the remaining models were compared using the DeLong test. ICH, intracranial hemorrhage; SDH, subdural hemorrhage; SAH, subarachnoid hemorrhage. *Standard error.

### 3.4 Comparison of the final weighted ensemble model with AI models that were previously built

**Figure 4:**
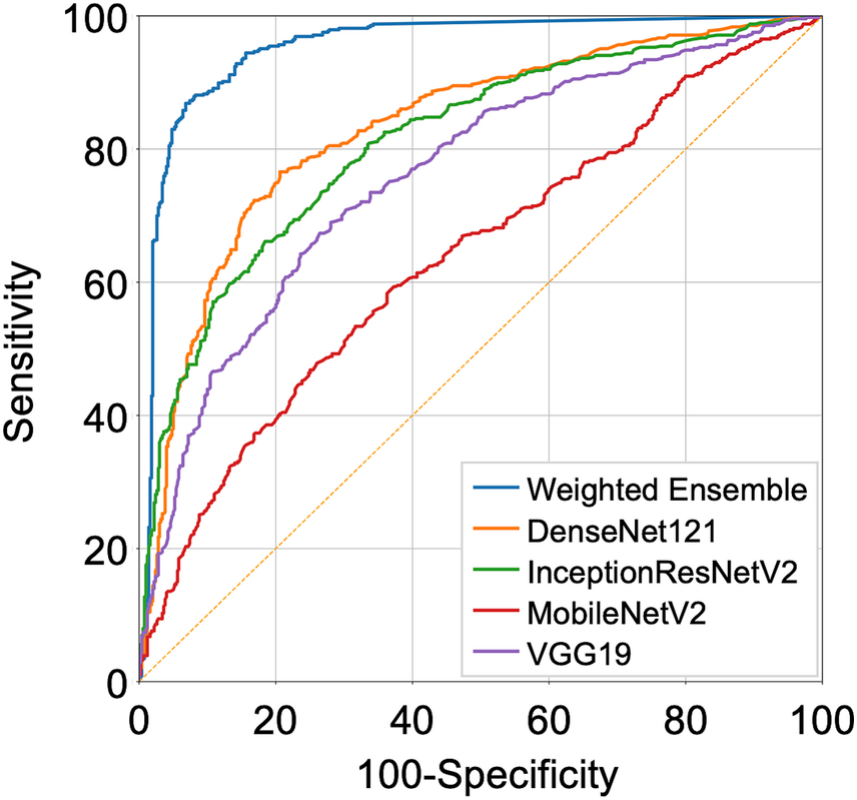
Receiver Operating Characteristic (ROC) curves representing the performance of the deep-learning models. DenseNet121, InceptionResNetV2, MobileNetV2, VGG19 trained on strongly annotated datasets and the final weighted ensemble model were applied to a test dataset combining the DUMC and Qure.ai datasets.

We compared the performance of the final weighted ensemble model with that of four well-known deep-learning models by using a test dataset that combines the DUMC and Qure.ai datasets. The weighted ensemble model significantly outperformed the other models in terms of sensitivity, specificity, and AUC (Figure 4 and Table 4, AUC [95% C.I.] of ensemble model, 0.953 [0.938–0.965]; InceptionResNetV2, 0.852 [0.828–0.873]; DenseNet121, 0.875 [0.852–0.895]; VGG19, 0.796 [0.770–0.821]; MobileNetV2, 0.650 [0.620–0.680]; *p* < 0.0001) Additional tests using either the DUMC or Qure.ai dataset showed similar results (Supplementary Figure S6).

**Table 4:** The performance of the weighted ensemble model and four well-known deep learning networks, DenseNet121, InceptionResNetV2, MobileNetV2, and VGG19 on a test dataset.

	Total dataset		
	AUC	Sensitivity	Specificity
**Ensemble model**	0.953 (0.938–0.965)	0.928 (0.901–0.949)	0.857 (0.824–0.887)
P for AUC difference	Reference		
**InceptionResNetV2**	0.852 (0.828–0.873)	0.802 (0.765–0.837)	0.715 (0.674–0.755)
P for AUC difference	*p* < 0.0001 (*0.0128)		
**DenseNet121**	0.875 (0.852–0.895)	0.856 (0.822–0.886)	0.709 (0.667–0.749)
P for AUC difference	*p* < 0.0001 (*0.0119)		
**VGG19**	0.796 (0.770–0.821)	0.751 (0.711–0.789)	0.705 (0.663–0.745)
P for AUC difference	*p* < 0.0001 (*0.0144)		
**MobileNetV2**	0.650 (0.620–0.680)	0.632 (0.588–0.675)	0.573 (0.528–0.617)
P for AUC difference	*p* < 0.0001 (*0.0178)		

The external test datasets were a composite of the DUMC and Qure.ai datasets containing 487 cases with ICH and 499 cases without hemorrhage. All AUCs derived from other deep learning models were significantly (DeLong test, all *p* < 0.001) lower than that of the Ensemble model. *Standard error.

### 3.5 Review of “difficult” 161 ICH cases

A total of 91 cases from the DUMC dataset were selected as difficult-for-AI cases: 17 cases from those classified as ICH that AI identified with a lesion probability of ≤0.6 and 74 cases from those classified as normal that AI identified with a lesion probability of ≥0.4 in external testing. A total of 70 cases from the Qure.ai datasets were selected as difficult-for-humans based on the three annotators’ disagreement. Six experts re-annotated these 161 cases for the presence vs. absence of ICH; there was complete agreement among the six experts for 81 cases, whereas there was at least one disagreement for 80 cases. For all 161 cases, the final weighted ensemble model showed an accuracy of 0.441, sensitivity of 0.431, and specificity of 0.462. The accuracies of the six experts were 0.671, 0.764, 0.708, 0.640, 0.683, and 0.714, with the interrater agreement (the Fleiss’ kappa value) being 0.536.

Among 95 cases where one or more experts disagreed with the initial ground truth, 80 were unanimous, five were disagreed upon by one expert, seven were disagreed upon by two experts, and three were disagreed upon by three experts. In 48 cases, the ground truth was changed following discussion and majority voting in the consensus meeting. The accuracy, sensitivity, and specificity of the weighted ensemble model for the revised ground truth was, respectively, 0.491, 0.457, and 0.525, without showing significantly differences when compared with those for the original ground truth. Supplementary Table S3 shows the qualitive assessments of the cases where the weighted ensemble model predicted incorrectly.

## 4 Discussion

This study demonstrated that (a) a deep-learning algorithm for detecting ICH trained with a strongly annotated dataset outperformed models trained with a weakly annotated dataset, and (b) a weighted ensemble model that integrated separate models trained using SDH, SAH, or small-lesion (≤ 5 mL) ICH datasets achieved a higher AUC than four previous deep-learning models on external testing.

Medical image segmentation requires a lot of labor and resources (25). Although many transfer learning methods for weakly or partially annotated data have been developed (26), there is still a need for large-scale annotated data. To the best of our knowledge, no deep-learning algorithm for ICH detection has been developed using large-scale CT scans with segmentation annotation. We found that the accuracy of three of the four previously reported deep-learning models improved after training with strongly annotated datasets, compared to weakly annotated datasets. Due to the large number of small ICH lesions or those with similar attenuation to normal tissue, it might be helpful to supervise the deep-learning model with the lesion’s location information. This could minimize the “location loss” of the saliency map, leading to better ICH detection.

Compared with magnetic resonance imaging, CT is less expensive, faster, and better at detecting ICH. However, some ICHs are more likely to be misdiagnosed due to their varied location and shape, small lesion size, and similar attenuation to adjacent tissue (13). For example, SDH an SAH are often difficult to distinguish from adjacent tissue (11), despite their distinct locations and shapes. Although there are classification methods for small lesions (27), a new deep learning strategy based on more comprehensive feature data may improve ICH detection performance. Weighted ensemble models can preserve the relative importance or performance of each base model by assigning different weights (28, 29). Song et al. (28) demonstrated improved model performance from heterogeneous multi-omics data by weighted ensemble, while preserving the local structure of each original sample feature obtained by different methods. Yin et al. (29) classified the amino acid sequence of influenza virus into eight segments and trained each model on each segment with ResNeXt, and developed a weighted ensemble model that showed improved performance. We achieved higher ICH detection accuracies using weighted ensemble models that combined multiple separate models trained with datasets specialized for SDH, SAH, or small lesions. Employing the weighted ensemble models, we also observed an increasing trend in specificity.

Despite recent development of many deep-learning algorithms for imaging diagnosis of ICH, their clinical application has yet to be accomplished. In addition to technical challenges such as the domain shift problem and the shortcut problem, there are also instances where determining the ground truth is difficult or inter-physician agreement is limited (30–32). After our expert meeting, as high as 30% (48/161) of difficult ICH cases, which however represented only 4.9% of the total test dataset cases (*n* = 986), required re-labeling of their ground truth. This may explain why it is challenging for AI to learn medical images that are difficult for experienced clinicians. Moreover, the re-labeling did not improve the accuracy of ICH detection by our weighted ensemble model trained with a total of 13,926 strongly annotated CT data. Further studies are required to investigate if fine-tuning a model after training with a larger high-quality training dataset, where difficult data are augmented, could increase the robustness, generalization, and discriminative power of the deep-learning algorithm.

Noisy labels have a negligible effect on the model performance in large datasets. In a handwritten number dataset, increasing the accuracy of random labels by only 1% significantly improved the model performance (33, 34). However, if difficult cases are mixed at a low frequency across a dataset, noisy labels may affect ICH detection. There is a high demand in the medical profession for a model that can accurately diagnose both easy and complex cases. False positives and negatives may result in unnecessary and missed therapy, respectively. Future research should investigate if (a) using weighted ensemble models and (b) expert review of difficult cases and adding them to a training dataset could overcome these challenges.

Our study has limitations. First, except for age and sex, no clinical information was available. The strongly annotated dataset consisted of patients from hospitals in South Korea only. Second, the classification and segmentation of some training data may not be accurate, because of the inclusion of difficult cases. Third, the accuracies for SDH and SAH cases were lower than the accuracy for all cases, although we showed comparable accuracies and higher specificities for them before and after applying the weighted ensemble model. In the test datasets, the “difficult” cases included a number of SDH and SAH cases. The SDHs and SAHs in the difficult cases had similar locations or attenuations to normal structures such as the falx, tentorium cerebelli, and venous sinus. Although we did not double-check the ground truth in the training dataset, the ground truth in the training dataset may not be 100% accurate. In future research, it may be necessary to establish the ground truth through expert opinion, especially in difficult cases. Fourth, it was not possible to compare the previously published AI algorithms for ICH detection to the same test dataset. Fifth, the proportions of mixed hemorrhages were high in the RSNA and AI-Hub datasets.

In conclusion, we developed a weighted ensemble model for ICH detection by training with strongly annotated CT scans obtained from multiple centers. Although challenging cases existed, external testing with a dataset from different ethnic origins demonstrated excellent performance of our model. We also showed that a better understanding and management of cases that are challenging for AI and humans is required to facilitate clinical use of ICH detection algorithms.

## Data availability statement

The raw data supporting the conclusions of this article will be made available by the authors, without undue reservation.

## Ethics statement

This study, which involved human subjects, was approved by the institutional review board of Dongguk University Medical Center (No. DUIH 2018-03-018) and JLK Inc (20220407-01). The study was conducted in accordance with the local legislation and institutional requirements. The ethics committee/institutional review board waived the requirement of written informed consent for participation from the participants or the participants’ legal guardians/next of kin due to data anonymization, inability to contact patients, and minimal risk.

## Author contributions

D-WK: Conceptualization, Data curation, Formal analysis, Investigation, Methodology, Project administration, Writing – original draft, Writing – review & editing. G-HP: Data curation, Formal analysis, Investigation, Methodology, Project administration, Software, Writing – original draft, Writing – review & editing. W-SR: Conceptualization, Data curation, Formal analysis, Investigation, Methodology, Project administration, Software, Supervision, Writing – original draft, Writing – review & editing. DS: Supervision, Writing – review & editing. MK: Formal analysis, Investigation, Writing – review & editing. YSK: Formal analysis, Investigation, Writing – review & editing. C-YP: Formal analysis, Investigation, Writing – review & editing. K-JL: Formal analysis, Investigation, Writing – review & editing. M-KH: Investigation, Supervision, Writing – review & editing. H-GJ: Formal analysis, Funding acquisition, Investigation, Methodology, Supervision, Writing – review & editing. D-EK: Formal analysis, Funding acquisition, Investigation, Methodology, Supervision, Writing – original draft, Writing – review & editing.

## Footnotes

^1^ https://aihub.or.kr/aidata/34101
