# Supplementary material for: Strengthening deep-learning models for intracranial hemorrhage detection: strongly annotated computed tomography images and model ensembles
Source: Front Neurol. 2023 Dec 29;14:1321964. doi: 10.3389/fneur.2023.1321964 (PMC10784380; doi:10.3389/fneur.2023.1321964)
Supplement: Supplementary file 1 [file Data_Sheet_1.pdf]

Supplementary Material

Supplementary Table S1. Summary of each deep-learning model used in this study.
Supplementary Table S2. Hyperparameters of skull stripping model (pre-processing) and each ensemble base model implemented in this study.
Supplementary Table S3. The cases that the weighted ensemble model mispredicted among the "difficult" ICH cases.
Supplementary Figure S1. Four-channel input data for model development.
Supplementary Figure S2. Model development using weakly and strongly annotated ICH datasets.
Supplementary Figure S3. Schematic of the deep-learning model architecture used in this study.
Supplementary Figure S4. Schematic of weight model.
Supplementary Figure S5. Receiver Operating Characteristic (ROC) curves represent the performance of four well-known deep-learning networks according to the training datasets.
Supplementary Figure S6. Receiver Operating Characteristic (ROC) curves representing the performance of the deep-learning models for each test dataset.

Supplementary Tables

<table>
<tr><th></th><th>Training dataset</th><th>Annotation</th><th>AI model</th><th>Test dataset</th><th>Prediction result</th><th>Comment</th></tr>
<tr><td colspan="7">Weakly vs. strongly annotated datasets</td></tr>
<tr><td>Weakly annotated</td><td>RSNA</td><td>Slice-wise subtype classification</td><td rowspan="3">DenseNet121
InceptionResNetV2
MobileNetV2
VGG19</td><td rowspan="3">DUMC and Qure.ai</td><td rowspan="3">Case-wise probability of ICH</td><td rowspan="3">Equally balanced number of slices per ICH subtype for each dataset</td></tr>
<tr><td>Weakly annotated</td><td>AI-Hub without location</td><td>Slice-wise subtype classification</td></tr>
<tr><td>Strongly annotated</td><td>AI-Hub with location</td><td>Slice-wise subtype classification and segmentation</td></tr>
<tr><td colspan="7">Development of the weighted ensemble model</td></tr>
<tr><td>Lesion segmentation model</td><td rowspan="5">AI-hub with location (strongly annotated)</td><td rowspan="5">Slice-wise subtype classification and segmentation</td><td rowspan="5">2D U-net with the Inception module + weighted ensemble</td><td rowspan="5">DUMC and Qure.ai</td><td rowspan="5">Case-wise probability of ICH</td><td rowspan="5">Balanced the number of normal and abnormal cases in each model</td></tr>
<tr><td>ICH subtype classification/ segmentation model</td></tr>
<tr><td>SDH classification/ segmentation model</td></tr>
<tr><td>SAH classification/ segmentation model</td></tr>
<tr><td>Small lesion (≤ 5 mL) segmentation model</td></tr>
<tr><td colspan="7">Comparison of the final weighted ensemble model with other AI models</td></tr>
<tr><td>Weighted ensemble model</td><td rowspan="2">AI-hub with location (strongly annotated)</td><td rowspan="2">Slice-wise subtype classification and segmentation</td><td>Weighted ensemble model</td><td rowspan="2">DUMC and Qure.ai</td><td rowspan="2">Case-wise probability of ICH</td><td rowspan="2"></td></tr>
<tr><td>Four AI models (DenseNet121, InceptionResNetV2, MobileNetV2, and VGG19)</td><td>DenseNet121
InceptionResNetV2
MobileNetV2
VGG19</td></tr>
</table>

Supplementary Table S1. Summary of each deep-learning model used in this study. We summarized the training dataset, annotation, AI model, test dataset, prediction result, and additional comment. RSNA, the Radiological Society of North America; DUMC, Dongguk University Medical Center; ICH, intracranial hemorrhage; SDH, subdural hemorrhage; SAH, subarachnoid hemorrhage.

Models	Hyperparameters
Skull Stripping Model	Inception Module Each Filters: 2, 4, 8, 16, 32<br>MaxPooling Pool Size: 3×3<br>Conv2DTranspose Each Filters: 8, 8, 4, 2<br>Conv2DTranspose Kernel Size: 4×4<br>Fully Connected Layer Filters: 64, 128, 256, 512
Lesion segmentation model	Inception Module Each Filters: 2, 4, 4, 8, 16, 16, 32<br>MaxPooling Pool Size: 3×3<br>Conv2DTranspose Each Filters: 8, 4, 2<br>Conv2DTranspose Kernel Size: 4×4<br>Fully Connected Layer Filters: 32, 64, 128
ICH subtype classification/ segmentation model	Inception Module Each Filters: 12, 24, 24, 48, 96, 96, 192<br>MaxPooling Pool Size: 3×3<br>Conv2DTranspose Each Filters: 32, 16, 8<br>Conv2DTranspose Kernel Size: 4×4<br>Fully Connected Layer Filters: 192, 384, 768
SDH classification/ segmentation model	Inception Module Each Filters: 8, 16, 16, 32, 64, 64, 128<br>MaxPooling Pool Size: 3×3<br>Conv2DTranspose Each Filters: 32, 16, 8<br>Conv2DTranspose Kernel Size: 4×4<br>Fully Connected Layer Filters: 128, 256, 512
SAH classification/ segmentation model	Inception Module Each Filters: 8, 16, 16, 32, 64, 64, 128<br>MaxPooling Pool Size: 3×3<br>Conv2DTranspose Each Filters: 32, 16, 8<br>Conv2DTranspose Kernel Size: 4×4<br>Fully Connected Layer Filters: 128, 256, 512
Small lesion (≤ 5 mL) segmentation model	Inception Module Each Filters: 8, 16, 16, 32, 64, 64, 128<br>MaxPooling Pool Size: 3×3<br>Conv2DTranspose Each Filters: 64, 32, 16<br>Conv2DTranspose Kernel Size: 4×4<br>Fully Connected Layer Filters: 128, 256, 512

Supplementary Table S2. Hyperparameters of skull stripping model (pre-processing) and each ensemble base model implemented in this study.

False positive cases		False negative cases	
**Normal tissue**		SDH	15
Around falx	10	Hemorrhagic transformation	7
Transverse sinus	6	SAH	4
Bone artifact	8	Medulla hemorrhage	1
Vertebral artery	2	Thalamic hemorrhage	1
Medulla	1	BG IPH	3
Tentorium	1	Pontine hemorrhage	1
BG calcification	2	Contusional hemorrhage	2
**Abnormal tissue**		Other IPH	3
Metallic artifact	3	Tentorial hemorrhage	1
Motion artifact	7		
skull defect-dura	6		
Meningioma/tumor	2		
**Total**	48	**Total**	38

Supplementary Table S3. The cases that the weighted ensemble model mispredicted among the "difficult" ICH cases. ICH, intracranial hemorrhage; BG, basal ganglia; SDH, subdural hematoma; SAH, subarachnoid hemorrhage; IPH, intraparenchymal hemorrhage.

Supplementary Figures

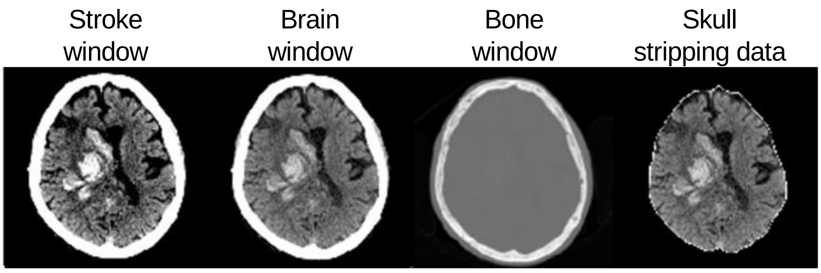

Supplementary Figure S1. Four-channel input data for model development. Stroke window (width 40 and level 40), brain window (width 80 and level 40), bone window (width 3,000 and level 500), and skull-stripped image.

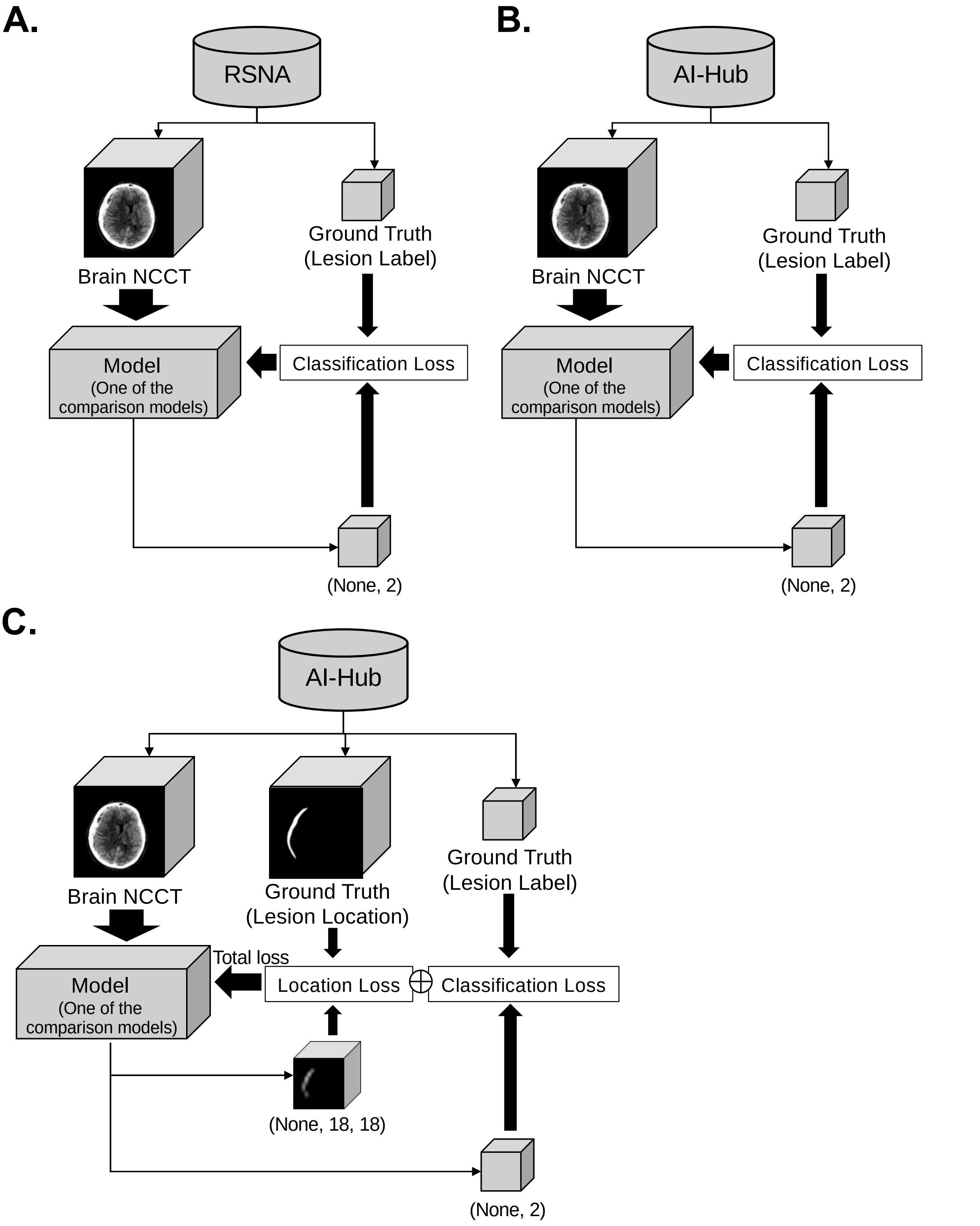

Supplementary Figure S2. Model development using weakly and strongly annotated ICH datasets. Training process using RSNA dataset (**A**), AI-Hub dataset without pixel-level annotations (**B**), and AI-Hub dataset with pixel-level annotations (**C**). For training with weakly annotated datasets **A** and **B**, we compared the model output slice-wise with ground-truth labeling to determine classification loss. For training with strongly annotated dataset **C**, we took the saliency map from the last convolutional layer and compared it to the ground-truth segmentation. We then calculated the "location loss" and "classification loss" and trained the model to minimize the summated loss. ICH, intracranial hemorrhage; RSNA, Radiological Society of North America; NCCT, noncontrast computed tomography.

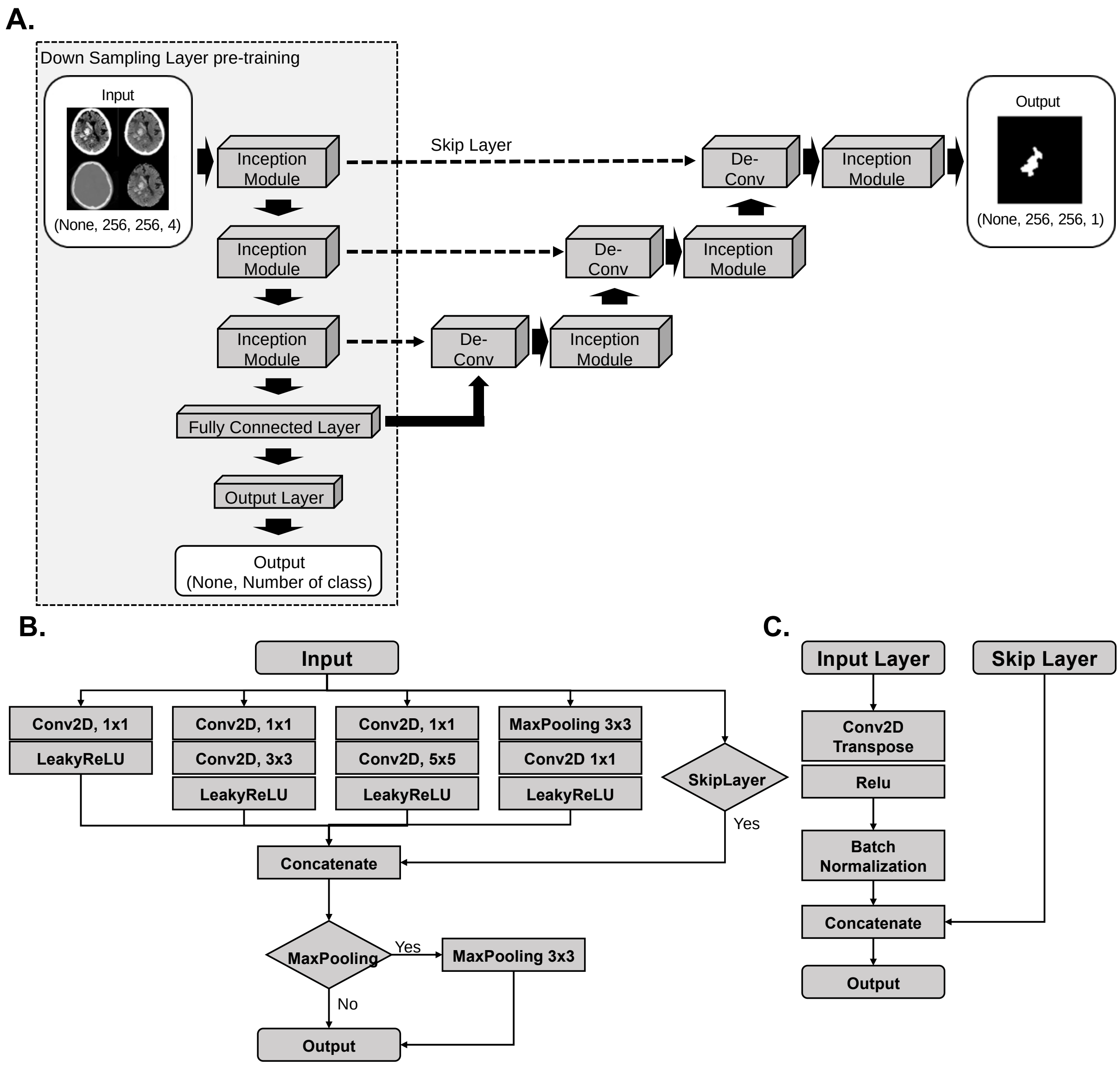

Supplementary Figure S3. Schematic of the deep-learning model architecture used in this study. A. Inception module-based U-Net model. During the encoding, Inception module was used. After convolved using various filter size, feature maps were concatenated and downsampled using a maxpooling. In the decoding phase, the deconvolution (upsampling) block (**C**) and Inception module were used. **B.** Detailed architecture of the inception module. **C.** Detailed architecture of deconvolution block. Details of hyperparameters were provided in Supplementary Table S2.

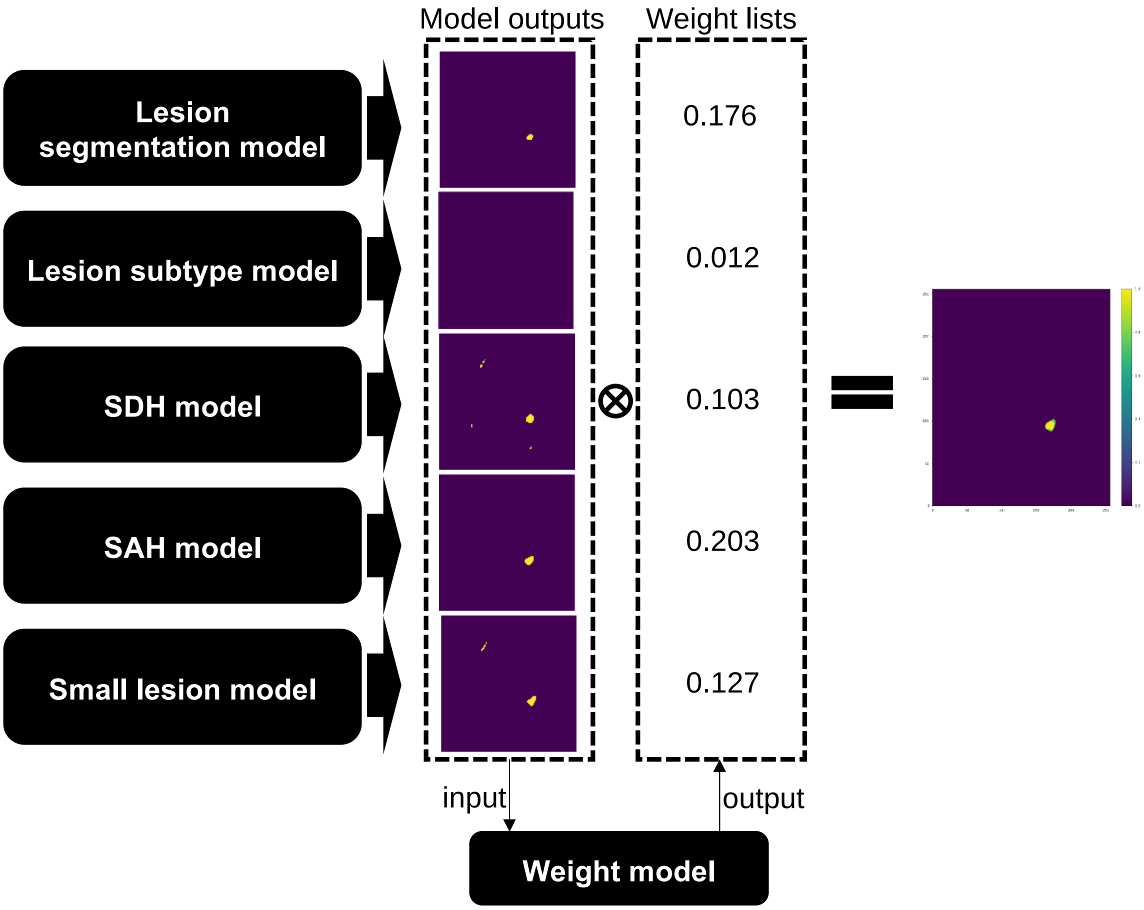

Supplementary Figure S4. Schematic of weight model. The model utilizes input data comprising the five-channel segmentation results obtained from the five base models. The final segmentation output is truncated at a threshold of 0.5. Each channel represents a probability value ranging from 0 to 1.

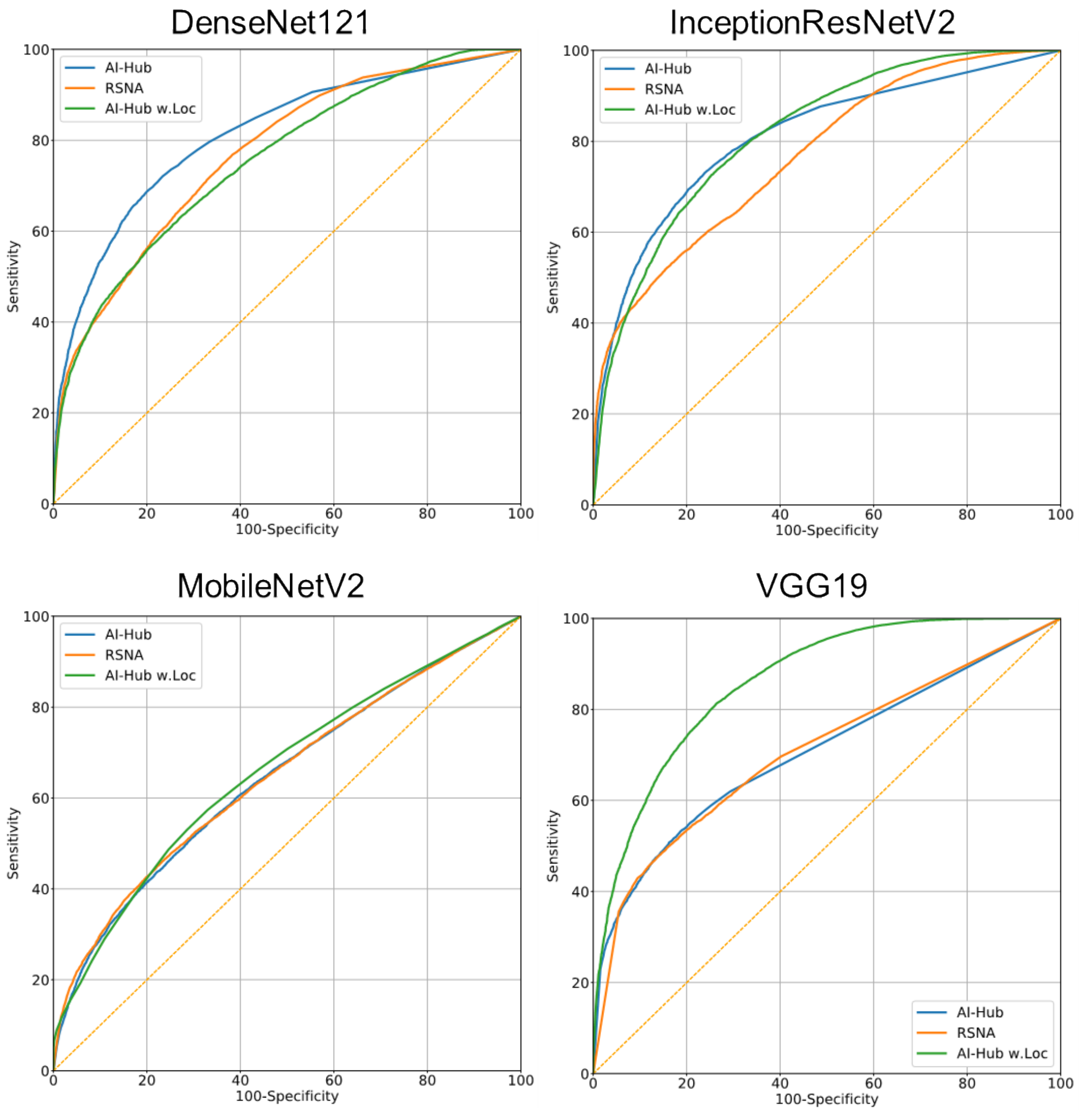

Supplementary Figure S5. Receiver Operating Characteristic (ROC) curves represent the performance of four well-known deep-learning networks according to the training datasets. The blue line indicates the AI-Hub dataset without location, the orange line indicates the RSNA dataset, and the green line indicates the AI-Hub dataset with location.

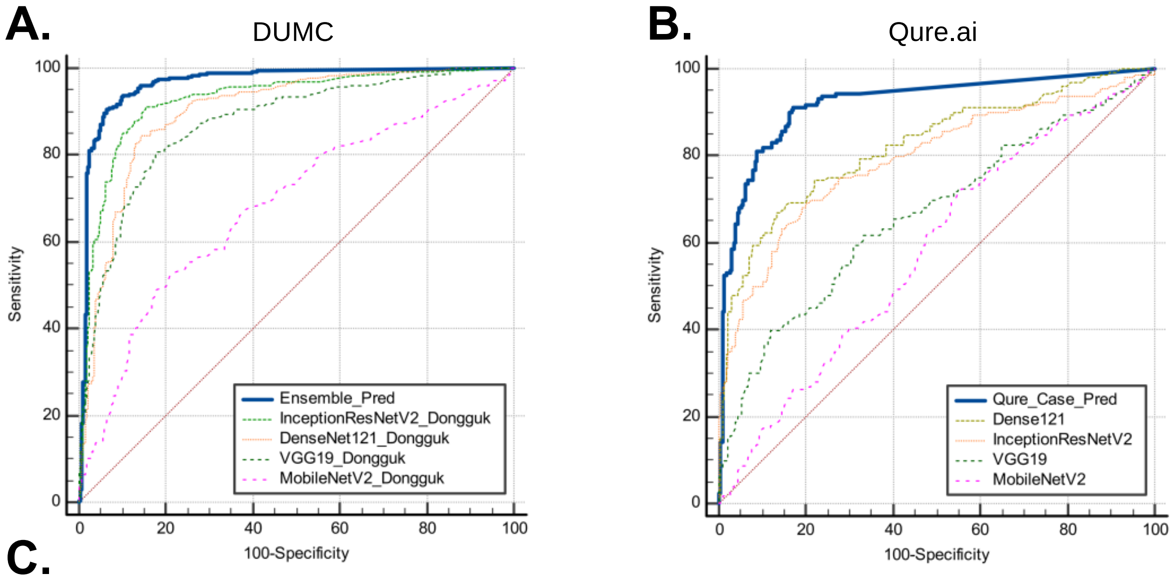

C.

95% C.I.	DUMC dataset			Qure.ai dataset		
	AUC*	Sensitivity	Specificity	AUC*	Sensitivity	Specificity
**Ensemble model**	0.964 (0.946 - 0.977)	0.929 (0.896 - 0.955)	0.864 (0.818 - 0.903)	0.937 (0.934 - 0.939)	0.948 (0.944 - 0.951)	0.914 (0.910 - 0.918)
**InceptionResNetV2**	0.926 (0.903 - 0.946)	0.908 (0.872 - 0.937)	0.846 (0.798 - 0.887)	0.793 (0.749 - 0.832)	0.750 (0.676 - 0.815)	0.721 (0.658 - 0.779)
**DenseNet121**	0.901 (0.874 - 0.923)	0.847 (0.803 - 0.884)	0.853 (0.806 - 0.893)	0.823 (0.782 - 0.860)	0.762 (0.689 - 0.826)	0.703 (0.639 - 0.762)
**VGG19**	0.872 (0.843 - 0.898)	0.807 (0.760 - 0.849)	0.824 (0.774 - 0.867)	0.665 (0.615 - 0.712)	0.619 (0.539 - 0.694)	0.668 (0.603 - 0.729)
**MobileNetV2**	0.685 (0.681 - 0.690)	0.608 (0.553 - 0.662)	0.655 (0.596 - 0.712)	0.580 (0.529 - 0.630)	0.637 (0.558 - 0.712)	0.504 (0.437 - 0.571)

Supplementary Figure S6. Receiver Operating Characteristic (ROC) curves representing the performance of the deep-learning models for each test dataset. A. DenseNet121, InceptionResNetV2, MobileNetV2, VGG19 trained on strongly annotated datasets and the final weighted ensemble model were applied to the DUMC (**A**) and Qure.ai (**B**) dataset. **C.** AUC, sensitivity, and specificity of each deep-learning model on DUMC and Qure.ai datasets. DUMC, Dongguk University Medical Center; AUC, area under the curve. *All AUCs derived from the other deep-learning models were significantly (DeLong test, all $P < 0.001$) lower than those of the ensemble model.
